# Prevalence of neutropenia in children by nationality

**DOI:** 10.1186/s12878-016-0054-8

**Published:** 2016-05-21

**Authors:** Srdjan Denic, Hassib Narchi, Lolowa A. Al Mekaini, Suleiman Al-Hammadi, Omar N. Al Jabri, Abdul-Kader Souid

**Affiliations:** Department of Medicine, College of Medicine and Health Sciences, United Arab Emirates University, PO Box 17666, Al-Ain, United Arab of Emirates; Department of Pediatrics, College of Medicine and Health Sciences, United Arab Emirates University, Al-Ain, United Arab of Emirates; Ambulatory Healthcare Services, Abu Dhabi, United Arab of Emirates

**Keywords:** Public health, Ethnicity, Monocyte count, Malaria hypothesis

## Abstract

**Background:**

A high prevalence of neutropenia has been reported in several ethnic groups amongst whom many healthy individuals with low neutrophil counts undergo unnecessary investigations. This study aims to ascertain the prevalence of neutropenia (NP) in a large cohort of children from North African, Middle Eastern, and Asian countries residing in the United Arab Emirates.

**Methods:**

Neutrophil counts of 26,542 children (one day to six years of age) from 86 countries were analyzed. The subjects were enrolled in the Well-Child-Care program of Ambulatory Health Services of Emirate of Abu Dhabi, United Arab Emirates. NP was defined as a neutrophil count <1.5 × 10^9^/L and severe NP <0.5 × 10^9^/L.

**Results:**

The neutrophil counts reached a nadir in the fourth week of life and changed slightly from the age of six-months to six-years. The frequency of NP was (from West-to-East): North African Arabs 15.4 %, Green Crescent Arabs 9.8 %, Peninsular Arabs 10.9 %, Iranians 3.1 %, Afghanis 2.5 %, Pakistanis 5.6 %, Indians 10.2 %, and Filipinos 7.3 %. The frequency of severe NP in North African Arabs (Sudanese) was 2.8 %, Green Crescent and Peninsular Arabs ≤1 %, Indians 1.5 %, and Filipinos 1.8 %. In 12,703 Emirati children, the frequency of NP was 10.6 % similar to their adult counterparts.

**Conclusion:**

The prevalence of childhood NP varied considerably by geoethnicity. Measures to prevent the inappropriate investigations of healthy children with benign neutropenia are proposed.

**Electronic supplementary material:**

The online version of this article (doi:10.1186/s12878-016-0054-8) contains supplementary material, which is available to authorized users.

## Background

Neutropenia (NP) is common amongst several ethnic groups from Africa and Asia [1–8]. A NP frequency of up to 30 % from Africa has been reported [1]. Among African Americans, its prevalence is 4.4 % [3]. In United Arab Emirates (UAE), 10.7 % of the native population has absolute neutrophil counts less than 1.5×10^9^/L [7]. The evidence suggests the inheritance of NP is autosomal dominant or co-dominant in people of Sudanese origin and among natives of Arabia [6, 7]. Molecular studies in some people of African ancestry, on the other hand, have shown a strong association between familial NP and the null Duffy genotype (Fy-/Fy-) and no association with the heterozygote (Fy-/Fy+) and wild-homozygote (Fy+/Fy+) genotypes, suggesting an autosomal recessive inheritance [9].

The benign nature of ethnic NP is based on reports of the absence of recurrent infections in such individuals [1–8]. However, many healthy individuals with low neutrophil counts often undergo unnecessary investigations to exclude pathologic NP. In addition, benign neutropenia often changes medical management such as delaying administration of myelosuppressants, premature stopping of drug therapy, postponing elective surgery and preventing recruitment into clinical trials [10–17]. This study ascertained the prevalence of NP in a large cohort of infants and children from North African, Middle Eastern and Asian countries who reside in UAE.

## Methods

### Study setting and population

This study was conducted in the Emirate of Abu Dhabi, UAE. The country’s population is eight million, of which 15 % are Emiratis (ethnically Arab) and the remaining 85 % are temporary foreign workers from numerous countries including the Indian subcontinent, the Middle East and North Africa. The study cohort comprised 26,542 infants and children. Their ages ranged from 1 day to 6 years. These children were registered in the Well-Child Care Program at Ambulatory Health Services (AHS) funded by the Health Authority of Abu Dhabi. The blood samples were obtained at the treating physician’s discretion between April 2008 and December 2013 at three hospitals (64 %), 26 outpatient AHS centers (19 %) or unidentified sites (17 %). Only one sample per child was used in the analysis. Written consent was not obtained as all blood counts were performed as part of the standard care.

### Complete blood count

The blood samples were collected in BD Vacutainer® spray-coated K2EDTA tubes. The samples were mixed by inversion, transported at 2-8 °C, and tested as soon as they arrived at the laboratory. Blood cell counts were determined using the Cell-Dyne Ruby analyzers (Abbott Laboratories, Illinois, USA). The laboratories run daily internal quality controls before running patient samples and participate in External Quality Assurance program through the College of American Pathologists Proficiency Testing.

### Definition of neutropenia and estimation of gene frequency

NP was considered mild, moderate and severe if the count was <1.5 × 10^9^/L, <1.0 × 10^9^/L and <0.5 × 10^9^/L, respectively. As the neutrophil count normally oscillates, some children with NP occasionally have >1.5 × 10^9^/L neutrophils. In a cross-sectional study, this fraction contributes to undetected (hidden) NP [7].

We hypothesized that severe NP in Emiratis was caused by a homozygote genotype and milder NP by a heterozygote genotype, i.e., inherited at a single locus with two alleles (*q* and *p* = 1-*q*). The native population of UAE is tribal, nearly half of the marriages are arranged between close cousins, and the mean coefficient of inbreeding in the population (*F*) is 0.022 [18–20]. Therefore, the frequency of the NP allele (*q*) in a large sample of Emiratis was determined using the Hardy-Weinberg equation adjusted for inbreeding. The frequency of homozygotes was *q*^2^(1-*F*) + *qF* and heterozygotes *2pq*(1-*F*) [21].

### Statistical analysis

As neutrophil counts did not follow a normal distribution (Shapiro-Wilk test *p* < 0.001), the non-parametric two-sample Wilcoxon rank-sum (Mann–Whitney) test was used to compare values between two categories, and Kruskal-Wallis rank test for three or more categories. The Spearman’s correlation test was used to analyze the relation between neutrophil and monocyte counts. For all analyses, two-tailed *p*-value of < 0.05 defined statistical significance. Other standard descriptive and statistical methods were used. One subject was removed because of an impossible value.

### Ethics approval

The study was approved by the Institutional Review Board of College of Medicine and Health Sciences – UAE University (#13/14).

## Results

The enrolled children represented 86 nationalities, of which 25,435 (96 %) were from 32 countries in North Africa, the Middle East and Asia. The largest group (12,073) was that of Emirati nationals.

**Fig. 1:**
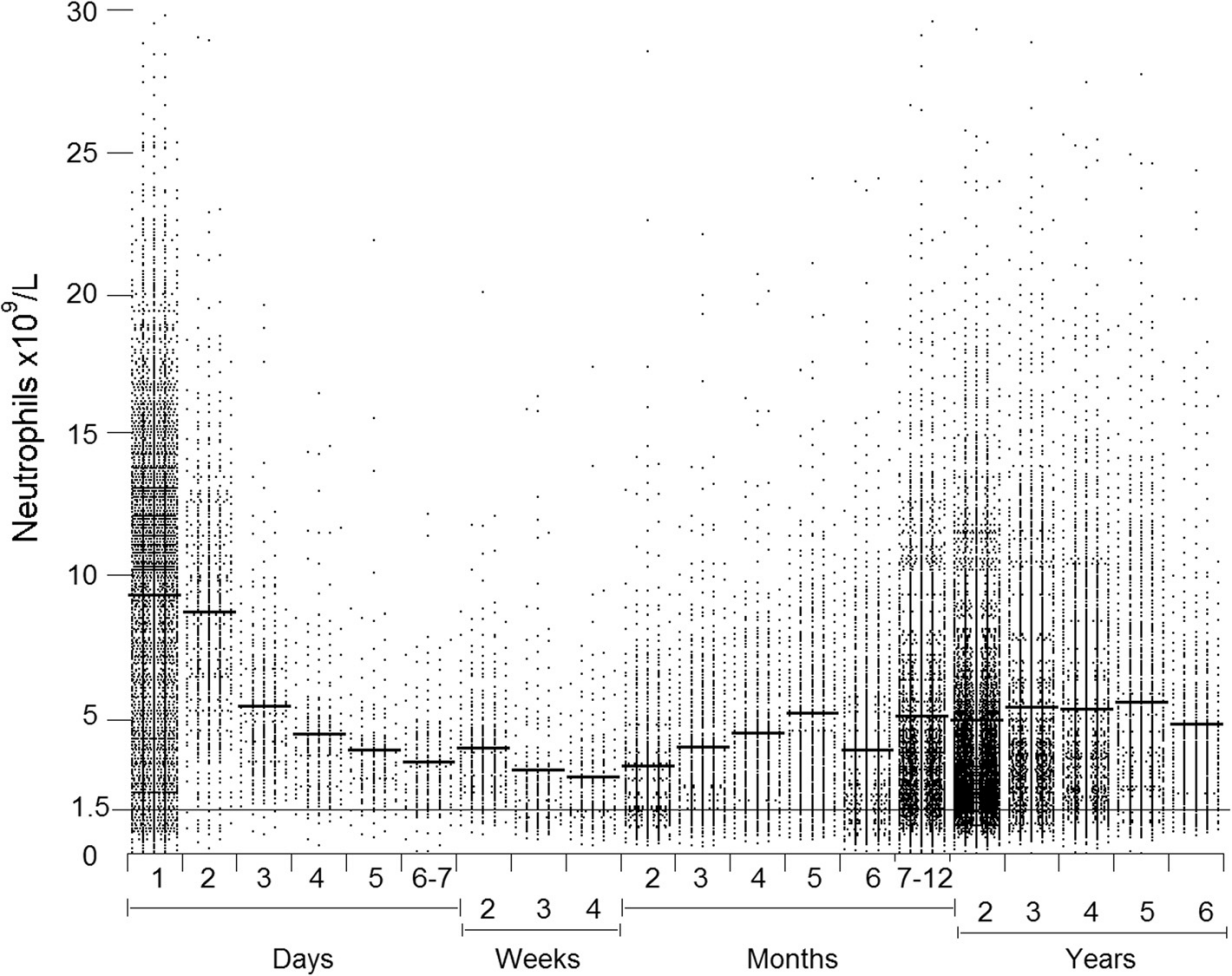
Neutrophil counts of the 26,542 children. Horizontal bars are means

Figure 1 shows the neutrophil counts by age (26,542 children from 86 nationalities). The counts decreased during the first week of life, reached a nadir in the fourth week and changed slightly between six-months and six-years. In the first month, the median (±SD) count (×10^9^/L) was lower (*p* ≤ 0.05) in 2,856 males (6.4 ± 4.9) than in 3,551 females (7.3 ± 5.6), but later in life the differences were insignificant (Table 1). The median, 10^th^ and 90^th^ percentile neutrophil count for eight Arab population is shown in Additional file 1: Figure S1.

**Table 1:** Median and 2.5^th^ percentile neutrophil counts of children from North Africa, Middle East and Asia

		Neutrophils × 10^9^/L							
		Days		Weeks	Months	Years			
		1	2–7	2–4	2–12	2	3	4	5–6
All	Median	8.7	5.1	2.7	3.3	3.4	3.7	3.8	3.8
	2.5^th^	0.9	1.3	0.8	0.7	0.8	0.9	0.9	1.0
		(3,360)	(1,429)	(615)	(6,997)	(5,846)	(3,097)	(2,463)	(2,470)
Females	Median	9.7	5.6	3.1	3.3	3.4	3.9	3.8	3.8
	2.5^th^	1.1	1.4	0.8	0.7	0.9	0.9	1.0	0.9
		(1,511)	(647)	(274)	(3,109)	(2,748)	(1,454)	(1,172)	(1,207)
Males	Median	8.0	4.7	2.5	3.3	3.4	3.7	3.8	3.7
	2.5^th^	0.9	1.1	0.8	0.7	0.8	0.9	0.9	1.0
		(1,849)	(782)	(341)	(3,888)	(3,098)	(1,643)	(1,291)	(1,263)
Outpatient	Median	-	-	-	2.5	2.7	3.2	3.4	3.6
	2.5^th^				0.5	0.7	0.8	0.9	0.9
					(1,544)	(2,905)	(1,812)	(1,673)	(2,021)
Inpatient	Median	8.7	5.1	2.8	3.6	4.8	4.9	4.9	5.0
	2.5^th^	0.9	1.3	0.8	0.8	1.0	1.0	1.2	1.1
		(3,360)	(1,425)	(591)	(5,453)	(2,941)	(1,285)	(790)	(449)

Values in parenthesis are number of children (only for *n* ≥50)

**Fig. 2:**
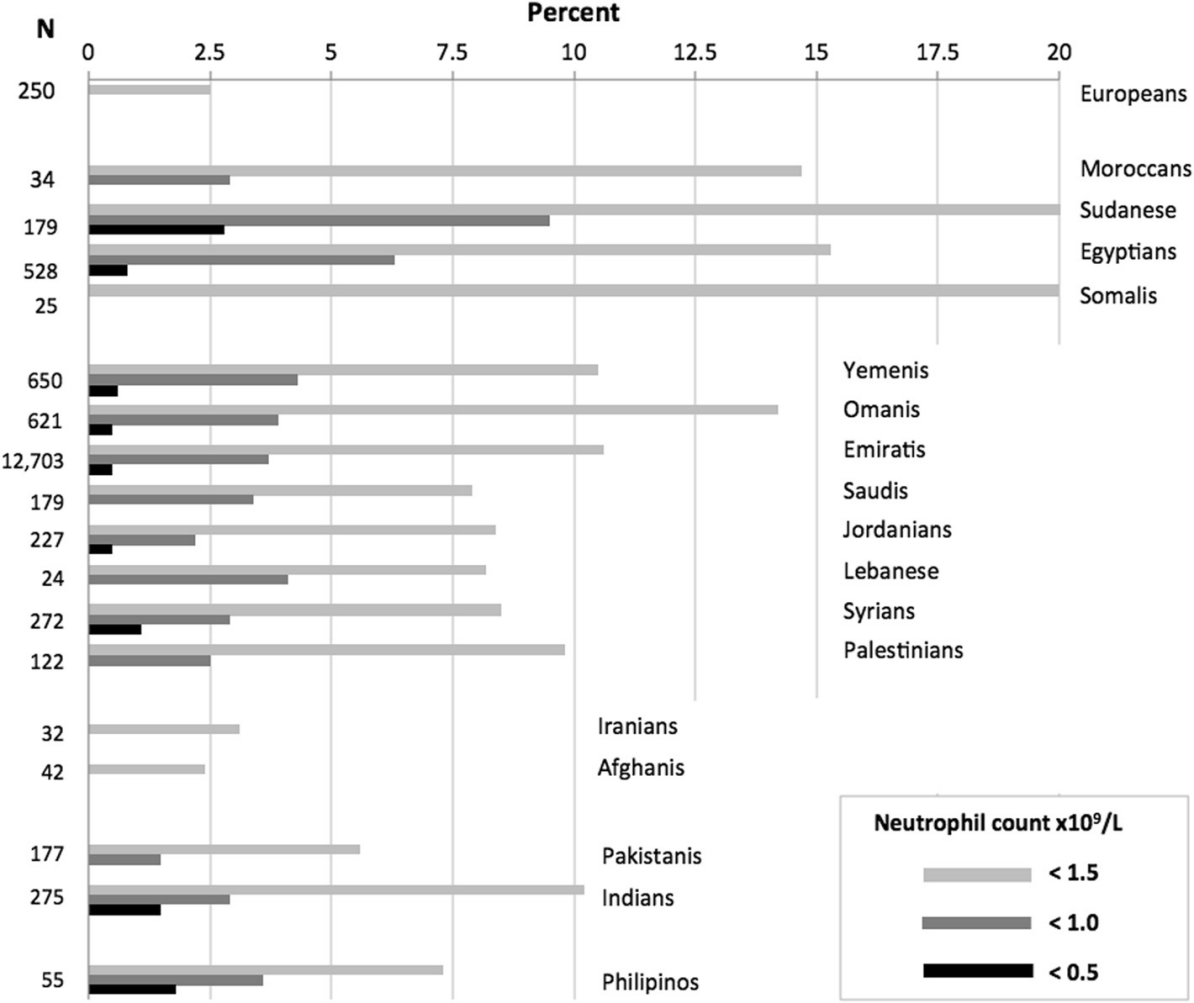
Prevalence of neutropenia in 0.5 to 6 year-old children by nationality. Data for Europeans are from reference [2]

Figure 2 shows the prevalence of NP by nationality in six-month to six-year-old children. Analysis of the neutrophil counts by geoethnicity is shown in Table 2. NP was most common amongst African Arabs (14.7 to 20.2 %); in these populations, the 2.5^th^ percentile count for 2-year-old children was 0.7×10^9^/L and for 5 to 6 year-old children, 1.1×10^9^/L. The frequency of NP in North Africans was 15.4 %, Peninsular Arabs 10.9 %, Indians 10.2 %, Green Crescent Arabs 9.8 %, Filipinos 7.3 %, Pakistanis 5.6 %, Iranians 3.1 %, and Afghanis 2.5 %. The prevalence of NP in Iranians and Afghanis was similar to that reported in Europeans [22–24]. The prevalence of severe NP in Sudanese was 2.8 %, Filipinos 1.8 %, Indians 1.5 %, and Middle Easterners ≤1 % (Fig. 2). The frequency of NP among eight Arab populations was not significantly different (see supplemental material).

**Table 2:** Median and 2.5^th^ percentile neutrophil counts of children by geo-ethnicity

		Neutrophils × 10^9^/L							
		Days		Weeks	Months	Years			
		1	2–7	2–4	2–12	2	3	4	5–6
North Africans	Median	5.8	4.6	-	2.7	2.6	3.0	3.1	3.8
	2.5^th^	0.9	0.9		0.5	0.7	0.7	0.6	0.9
		(171)	(84)		(252)	(363)	(132)	(104)	(108)
Peninsular Arabs	Median	9.0	5.3	2.7	3.4	3.6	3.8	3.8	3.5
	2.5^th^	1.0	1.3	0.8	0.7	0.8	1.0	1.0	1.0
		(2,585)	(969)	(529)	(5,929)	(4,782)	(2,187)	(1,399)	(776)
						2 – 6			
Green Crescent Arabs	Median	7.2	5.6	-	3.2	3.3			
	2.5^th^	0.7	1.4		0.9	1.0			
		(178)	(95)		(239)	(880)			
Iranians & Afghanis	Median	-	-	-	-	3.9			
	2.5^th^					1.4			
						(57)			
Indian Subcontinent	Median	7.5	5.1	-	3.2	3.2			
	2.5^th^	0.9	1.9		0.7	1.1			
		(180)	(98)		(211)	(510)			
Filipinos	Median	-	-	-	-	2.8			
	2.5^th^					1.4			
						(51)			

Values in parenthesis are number of children (only for *n* ≥50)

North Africans (1,243): Egyptians (771), Sudanese (278), Moroccan (67), Somalis (44), Mauritanians (39), Tunisians (20), Ethiopians (10), Algerians (7), Eritreans (4), Libyans (2)

Peninsular Arabs (21,883): Emiratis (19,545), Yemenis (1,058), Omanis (952), Saudis (292), Qataris (18), Bahrainis (7), Bedouins (6), Kuwaitis (5)

Green Crescent Arabs (1,119): Syrians (448), Jordanians (365), Palestinians (222), Lebanese (51), Iraqis (33)

Indian Subcontinent (1,042): Indians (490), Pakistanis (448), Bangladeshis (82), Sri Lankans (16), Nepalese (6)

We attempted to confirm the previously reported correlation between the monocyte and the neutrophil counts [7]. We found that the Spearman’s correlation coefficients between neutrophil and monocyte counts in the four largest ethnic groups were as follows: Egyptians, 0.46; Sudanese, 0.51; Emiratis, 0.44; and Indians, 0.47.

In the 12,703 Emirati children (aged six-months to six-years), the prevalence of NP (Fig. 2) was 10.6 %, similar to that of healthy adult Emiratis (10.7 %) [7]. The prevalence of severe NP of the children in this study was 0.53 %. For this sub-group we estimated frequency of NP allele which was 6.3 %, while the frequency of phenotype-derived heterozygosity was 11.6 %. The latter value was 1.51 % higher than the milder NP (10.7 % – 0.53 % = 10.07 %) observed in the studied population. This 1.51 % discrepancy in the frequency is explained by hidden NP.

## Discussion

This study confirms that NP is common in people from North Africa, the Middle East and South Asia (Fig. 2 and Table 2). The neutrophil counts were highest at birth, decreased in the first seven days of life, and reached a nadir in the fourth week of life. Thereafter, the counts increased until 6 months of age and changed a little from six months to six years (Fig. 1). This pattern is similar to that in European children [22–24]. The median neutrophil count was lower in male neonates than female neonates (Table 1). In one study of adult Africans, males had lower neutrophil counts than females [16].

People from North African countries have the highest frequency of NP, ranging from 14.7 to 20.7 % (Fig. 2). Benign NP was first reported from Africa and from countries to which Africans have migrated [2, 3, 25]. A high prevalence of NP was found in the Sudanese who migrated to the Middle East, and amongst Yemenis and Ethiopian Jews who might have acquired the trait from neighboring African populations [6, 8]. This study shows that 10.5 % of Yemenis and 20 % children from the neighboring Somalia have NP. The frequency of NP in Peninsular Arabs ranged from 7.9 to 14.2 %, and Green Crescent Middle East residents from 8.2 to 9.8 % (Fig. 2). The Duffy negative blood group, strongly associated with benign NP in societies of African ancestry, is also common among Arab populations [26]. This finding is consistent with the historical mixing of Arabs with native African populations suggesting a common origin. In the geographically more distant Iranians and Afghanis, the frequency of NP is considerably lower (3.1 and 2.5 %, respectively) being similar to Europeans (<2.5 %) [23]. Furthermore, Iranians, Afghanis and Europeans share common pre-historic origins. In the Indian subcontinent, frequency of NP increased from 5.6 % in Pakistanis (closer to Afghanis) to 10.2 % in Indians.

This geoethnic distribution of frequencies raises a possibility that Afro-Arab and Indian NP have independent origins (involving the same or different genetic mutations). This hypothesis is supported by the absence of associations with the Duffy negative genotype (a biomarker for African NP) among Indians [9, 27]. High prevalence of NP is also found in Filipinos (Fig. 2); historically, this nation has received migrants from the Indian subcontinent and does not have the Duffy negative genotype [28].

Considerable number of children have neutrophil counts <0.5 ×10^9^/L (2.8 % of Sudanese, 1.8 % of Filipinos and 1.5 % of Egyptians and Indians). A different genetic mutation could account for this severe NP phenotype. On the other side, in pedigree analyses in earlier study, consanguineous parents of offspring with severe NP have milder phenotype, suggesting a co-dominant inheritance [7]. Severe NP, thus, could represent a homozygote genotype and milder NP (nearly always >0.8 ×10^9^/L) a heterozygote genotype. This mode of inheritance is supported by our estimated frequency of presumed heterozygote (2*pq*, milder *plus* hidden phenotype) derived from frequency of severe NP (presumed homozygote, *q*^2^) in 12,703 Emiratis. In over dozen kinship groups (data not shown), the frequency of both phenotypes correlates (*r* = 0.468), providing additional support that one mutation is a cause of benign NP. However, other genes and polymorphisms are known to impact neutrophil production and more than one gene could be involved in benign NP [29]. A possible effect of environmental factors that could affect gene expression could not be excluded.

In studied populations, two observations support the benign nature of NP. The prevalence of NP in 12,703 Emirati children (10.6 %) is similar to 10.7 % in 1,032 healthy adult Emiratis [7]. This unchanged frequency, over an estimated18-year-period, is an epidemiological evidence of its benign nature. The positive correlation between the monocyte and neutrophil counts in four large ethnic groups (Egyptians, Sudanese, Emiratis and Indians) supports benign rather than secondary NP, in which monocytosis is a more common finding [30–32]. In general, our findings agree with earlier reports of a high frequency of benign NP in children from Sudan and Jordan [4, 6].

The “malaria hypothesis” proposed as an explanation of high prevalence of benign NP is based on (i) a long history of endemic malaria in populations which have high prevalence of NP and (ii) its associated monocytopenia which is linked with a less intense phagocyte-mediated inflammation [7, 33–35]. The finding of low prevalence of NP among Iranians and Afghanis (whose ancestral population recently migrated from the north to this region) and a high prevalence among people from Indian subcontinent and Philippines (in whom NP has not been previously reported) supports the hypothesis (Fig. 2). In addition, the low monocyte count we found in four ethnic groups with NP (correlation coefficients, 0.44 to 0.51) supports the assertion of the benign nature of the observed NP and, indirectly, the “malaria hypothesis.”

An important clinical implication of this study is that our suggested cutoff for NP (2.5^th^ percentile) in African Arabs should be 0.9 × 10^9^/L, in Middle Easterners 1.0 × 10^9^/L, and in Indians 1.1 × 10^9^/L (Table 2). In healthy adult Emiratis and adult Africans, this threshold should be 1.0 × 10^9^/L and 0.9 × 10^9^/L, respectively [7, 16]. These numbers are considerable lower than the commonly used NP cutoff of 1.5 × 10^9^/L. Consequently, healthy individuals from these regions with neutrophil counts <1.5 × 10^9^/L often undergo unnecessary investigations and inappropriate treatment [15, 17]. This problem could be addressed in several ways: (i) issuing ‘benign NP health cards’ to identify subjects with NP; (ii) creating a national electronic registry for NP; (iii) the screening for NP by adding leucocyte differential count to the screening tests for hemoglobinopathies already performed in many countries, and (iv) developing ethnic/nation-specific neutrophil reference values. In addition, general guidelines for investigating apparently healthy neutropenic subjects could improve the quality of health care delivery and lower health care costs. In conclusion, at least 137 million of the consolidated 1.8 billion children and adults of the 16 countries represented in this study (Additional file 1: Table S1) could benefit from higher awareness of physicians about benign neutropenia, preventing investigation and management pitfalls in neutropenic patients.

## Additional file

Additional file 1: Figure S1. Neutrophil count distribution of 14,796 children from eight Arab populations. **Table S1.** Estimated number of children and adults with neutropenia in 16 North African, Middle Eastern and Asian countries. (DOCX 255 kb)
