# Supplementary material for: Prevalence of neutropenia in children by nationality
Source: BMC Hematol. 2016 May 21;16:15. doi: 10.1186/s12878-016-0054-8 (PMC4875641; doi:10.1186/s12878-016-0054-8)
Supplement: Additional file 1: Figure S1. — Neutrophil count distribution of 14,796 children from eight Arab populations. Table S1. Estimated number of children and adults with neutropenia in 16 North African, Middle Eastern and Asian countries. (DOCX 255 kb) [file 12878_2016_54_MOESM1_ESM.docx]

**Additional file 1**

**Prevalence of neutropenia in children by nationality**

Srdjan Denic^1,*^, Hassib Narchi^2^, Lolowa A. Al Mekaini^2^, Suleiman Al Hammadi^2^,

Omar N. Al Jabri^3^, Abdul-Kader Souid^2^

^1^Department of Medicine, College of Medicine and Health Sciences, United Arab Emirates University, Al-Ain, UAE. ^2^Department of Pediatrics, College of Medicine and Health Sciences, United Arab Emirates University, Al-Ain, UAE.  ^3^Ambulatory Healthcare Services, Abu Dhabi, UAE.

**Supplemental Results**

The differences in the frequency of neutropenia (NP) among eight Arab populations (Emirati, Jordanians, Lebanese, Omani, Palestinians, Saudi, Syrians and Yemeni) in Figure 2 were evaluated using Chi squared test and Fisher’s exact test for values ≤ 5. The frequencies of overall NP [absolute neutrophil count (ANC) < 1.5x10^9^/L], moderate NP (ANC<1.0x10^9^/L) and severe NP (ANC<0.5x10^9^/L) were not different, p=0.09, p=0.8, and p=0.8, respectively.

**Supplemental Figure**

**
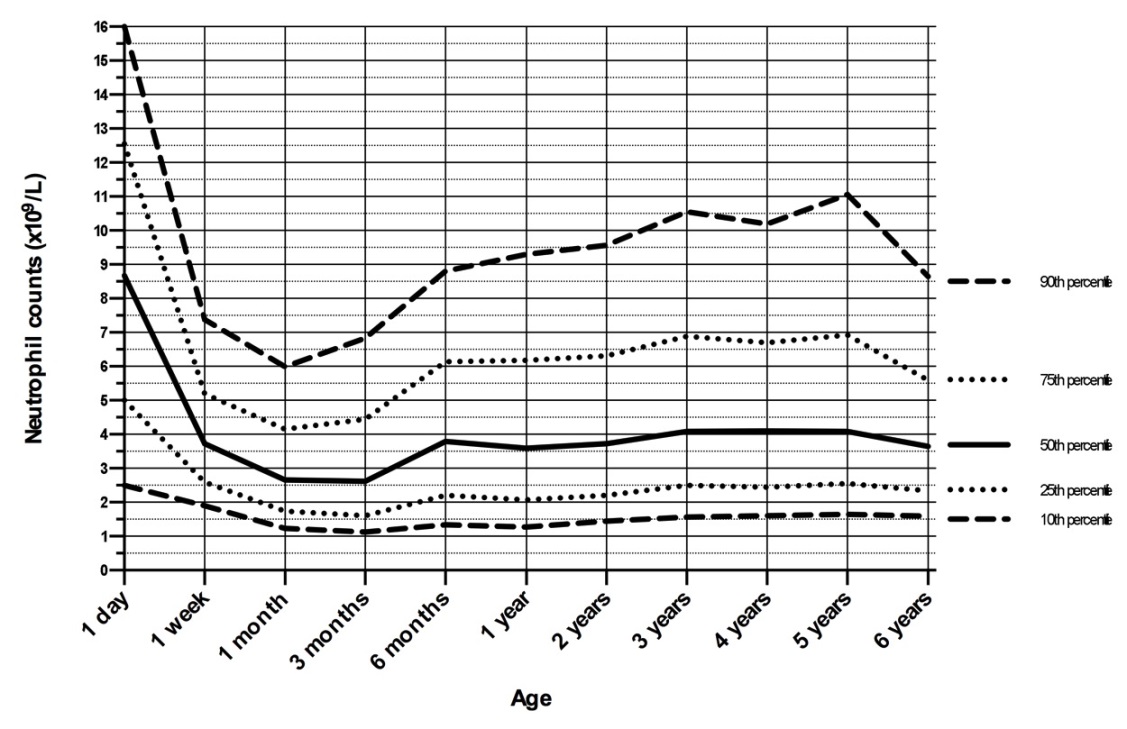
**

**Figure S1**. Neutrophil count distribution of 14,796 children from eight Arab populations.

**Supplemental Table**

**Table S1. Estimated number of children and adults with neutropenia in 16 North African, Middle Eastern and Asian countries**

Data on population from CIA The World Fact Book for 2014.
